# Complications of Evans' syndrome in an infant with hereditary spherocytosis: a case report

**DOI:** 10.1186/1756-8722-2-40

**Published:** 2009-09-10

**Authors:** Hideki Yoshida, Hiroyuki Ishida, Takao Yoshihara, Takashi Oyamada, Masataka Kuwana, Toshihiko Imamura, Akira Morimoto

**Affiliations:** 1 Department of Pediatrics, Matsushita Memorial Hospital, Moriguchi, Japan; 2 Department of Forensic Medicine, Jichi medical University, Tochigi, Japan; 3 Division of Rheumatology, Department of Internal Medicine, Keio University, Tokyo, Japan; 4 Department of Pediatrics, Kyoto Prefectural University of Medicine, Kyoto, Japan

## Abstract

Hereditary spherocytosis (HS) is a genetic disorder of the red blood cell membrane clinically characterized by anemia, jaundice and splenomegaly. Evans' syndrome is a clinical syndrome characterized by autoimmune hemolytic anemia (AIHA) accompanied by immune thrombocytopenic purpura (ITP). It results from a malfunction of the immune system that produces multiple autoantibodies targeting at least red blood cells and platelets. HS and Evans' syndrome have different mechanisms of pathophysiology one another. We reported the quite rare case of an infant who had these diseases concurrently. Possible explanations of the unexpected complication are discussed.

## Background

Hereditary spherocytosis (HS) is caused by a variety of molecular defects of erythrocyte membrane proteins. These proteins are necessary to maintain the normal shape of erythrocytes. As the spleen normally targets abnormal shaped red blood cells (RBCs), it also destroys spherocytes. Autoimmune hemolytic anemia (AIHA) is the most common autoimmune hemolytic diseases. The RBC attached antibodies was recognized and grabbed onto by macrophages in the spleen. These cells will pick off portions of RBC membrane, that causes spherocytic change. Spherocytes are not as flexible as normal shaped RBCs, and will be singled-out for destruction in the reticuloendothelial system, that gives rise to extravascular hemolysis [1]. Immune thrombocytopenic purpura (ITP) is a condition of having a low platelet count caused by autoimmune with antibodies against platelets. The coating of platelets with antibodies renders them susceptible to opsonization and phagocytosis by splenic macrophages [2]. Evans' syndrome refers to a major disorder in immunoregulation characterized by AIHA accompanied by ITP [3]. HS and Evans' syndrome have different mechanisms of pathophysiology one another. Herein, we report the first case confirmed Evans' syndrome associated with HS.

## Case presentation

The patient was born at 40 weeks' gestation with 2860 g by normal spontaneous vaginal delivery after an uncomplicated pregnancy. In family history, his mother underwent splenectomy due to controlling HS when she was 14 years old. At the age of 2 days, he had remarkable jaundice without hepatosplenomegaly. Blood chemical values were as follows: white blood cell (WBC) counts of 17,800/μl, RBC counts of 5,020 × 10^3^/μl, hemoglobin 18.0 g/dl, reticulocyte 8.0%, platelet count 305 × 10^3^/μl, total bilirubin 19.6 mg/dl. His and his mother's blood group (A, Rh+) were compatible, and his direct anti-globulin test (DAT) was negative. His erythrocytes showed high osmotic fragility in erythroresistant test (Table 1). Consequently, we diagnosed him with HS. He immediately received exchange transfusion for hyperbilirubinemia. He discharged at 6 days later with no complication. However, his hemoglobin gradually decreased (less than 7 g/dl) after leaving our hospital, and erythrocyte transfusion was needed. Steroid (betamethasone: 0.05 mg/kg) was given to him for suppressing the splenic function. As a result, his hemoglobin kept 8 to 9 g/dl without transfusion. During tapering a dosage of betamethasone, his platelet counts, but not other blood cell count, had suddenly decreased (57 × 10^3^/μl) at the age of 6 months. At this time, platelet-associated immunoglobulin (PAIgG) was high (239.0 ng/10^7 ^cell). Bone marrow examination revealed normal cellularity with increasing of megakaryocytes (305/μl). Increment of abnormal blasts, hemophagocytes and dysplastic cells were not found on bone marrow film. IgM-antibodies against cytomegalovirus, human immunodeficiency virus antibody, anti-nuclear antibody or anti-DNA antibody was not detected. He had no clinical feature, which suggested collagen disease or the coexistence of infectious diseases (Table 1).

**Table 1:** Laboratory findings

	**at 2 days**	**at 6 months**		**at 2 days**
WBC	17,200	13,600/μl	TSH	7.72 mU/ml
neut	74	64%	FT3	6.1 pg/ml
lym	13	32.5%	FT4	4.26 ng/dl
mono	12	1.5%	blood group	A, Rh (+)
baso	0	0%	Direct anti-globulin test	negative
eos	0.5	2%	Indirect anti-globuin test	negative
RBC	5020 × 10^3^	2840 × 10^3^/μl	eluate test	negative
Ret	8.0	18.4%	osmotic fragility in erythroresistant test	
Hb	18	8.6 g/dl	(after leaving 24 hours)	
Ht	53	24.9%	osmotic pressure starting hemolysis	>0.50% normal saline
Plt	305 × 10^3^	57 × 10^3^/μl	osmotic pressure finishing hemolysis	0.42% normal saline
T-Bil	19.6	4.6 mg/dl		at 6 months
D-Bil	1.6	mg/dl	C3	107 mg/dl
AST	53	32 IU/l	C4	24 mg/dl
ALT	9	18 IU/l	anti nuclear Ab	<40
LDH	797	340 IU/l	anti-DNA Ab	<2.0 IU/ml
Alp	302	723 IU/l	anti-cytomegalovirus IgM	0.58 (EIA)
TP	5.8	6.7 g/dl	anti-parvo B-19 IgM	0.32 (EIA)
Alb	3.5	4.8 g/dl	PAIgG	239 ng/10^7 ^cells
BUN	7	5 mg/dl	Direct anti-globulin test (2nd times)	negative
Cre	0.64	0.19 mg/dl	Indirect anti-globuin test (2nd times)	negative
CRP	0.19	0.21 mg/dl	Bone marrow examination	
			nucleated cell count	310 × 10^3^/μl
			megakaryocyte	305/μl
			abnormal blast	not found
			phagocyte	not found
			dysplasia	not found

**Figure 1:**
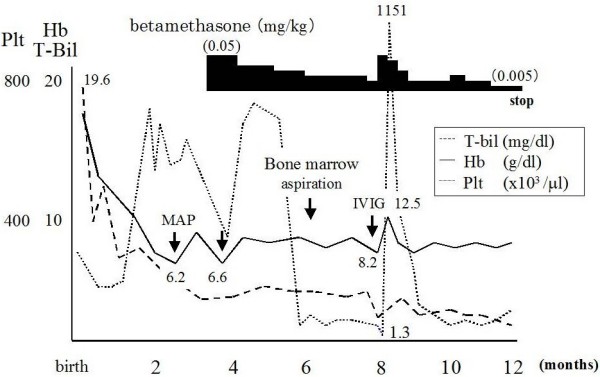
**Clinical course**. Evans' syndrome in our patient was developed when corticosteroid used for suppressing the splenic function was tapered. Following a high dose of intravenous immunoglobulin and a dosage of steroid, not only platelet counts but also hemoglobin levels quickly increased in association with decrement in reticulocytes and total bilirubin.

At the age of 8 months he had purpura and gingival bleeding following a cold. Although WBC counts (8,300/μl) and hemoglobin levels (8.3 g/dl) were unchanged, platelet counts progressively decreased (13 × 10^3^/μl) again. Because a complication of ITP was most suspected, intravenous immunoglobulin (IVIG) (1 g/kg) and a dosage of steroid were administered to him. Unexpectedly, not only platelet counts (from 13 to 1017 × 10^3^/μl 2 weeks later) but also hemoglobin levels (from 8.6 to 12.5 g/dl) quickly increased in association with decrement in reticulocytes and total bilirubin (from 626 to 229 × 10^3^/μl, from 4.5 to 1.9 mg/dl, respectively) in response to IVIG therapy. At that time, the percentage of reticulated platelets was 1.3% (reference value: <2%), and a level of thrombopoietin was normal (32 pg/mL, reference value: <142 pg/ml). Upshaw Schulman syndrome was excluded because of only slight low level of ADAMTS-13 activity (34.7%, reference value: 70-130%) and normal result of von Willebrand factor multimer analysis [4]. Because the erythrocyte binding IgG quantitative analysis showed mild elevation in the patient, we concluded that the infant with HS was accompanied by ITP and DAT negative AIHA (Evans' syndrome). At the age of 10 months after confirming stability of platelet counts, tapering betamethasone resulted in gradually decreasing hemoglobin levels and platelet counts (hemoglobin 9 g/dl, platelets 3-5 × 10^3^/μl) as Figure 1 shown.

## Methods & results

**Figure 2:**
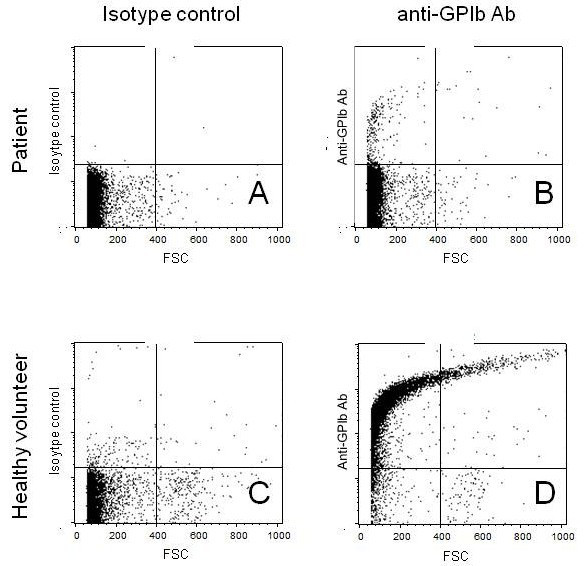
**GPIb detection on platelets from normal volunteer and our patient by flow cytometric analysis**. Platelets from our patients were negative against FITC-conjugated anti-GPIb antibody, suggesting that they were already coated with acquired anti-GPIb antibody.

We examined platelet specific autoantibodies, and erythrocyte binding IgG quantitative analysis based on the methods as previously described[5,6]. Although a level of platelet-adhering GPIIb/IIIa antibody slightly increased (4.1 U/10^6 ^cells, reference value: <3.3 U), the number of GPIIb/IIIa antibody-producing B cells analyzed with enzyme-linked immunospot (ELISPOT) assay was normal (0.2/10^5 ^peripheral blood mononucleated cells (PBMC), reference value: <2.0/10^5 ^PBMC). With flow cytometric analysis anti-GPIb, corresponding to CD42b, was not clearly dyed on the patient platelets (Figure. 2), suggesting that the existence of autoantibody adhering to GPIb on the platelets. Taken together, ITP caused by GPIb antibody but not GPIIb/IIIa was suggested. The erythrocyte binding IgG quantitative analysis showed mild elevation in the patient (218 IgG-molecule/RBC (reference value: 33 ± 13)), indicating he had DAT-negative AIHA [7].

## Discussion

We reported a case of 6 month-old infant affected by HS, accompanied by Evans' syndrome. The diagnosis of HS was made by family history (his mother had already been diagnoed with HS), a negative DAT, high osmotic fragility in erythroresistant test and later typical spherocytic morphology.

Platelet surface GPIIb/IIIa and GPIb are the most common antigenic targets in ITP [8]. No increasing of the number of B cells producing GPIIb/IIIa antibody in the patient peripheral blood with ELISPOT assay and the unbinding of anti-GPIb antibody to the patient's platelets with flow cytometric analysis suggested that autoantibody adhering to GPIb on the platelets was responsible for thrombocytopenia.

The hallmark of AIHA is a positive DAT by which IgG and/or complement are found on the RBC surface. However, the incidence of a negative DAT in patients with AIHA has been reported to be between 2 to 4% [9]. Explanation for the negative DAT in some patients with AIHA is that the number of IgG molecules on RBC necessary for accelerated *in vivo *destruction is sometimes lower than the number of that to yield a positive DAT. Because in our patient anemia improved with a decrement of bilirubin following IVIG and an erythrocyte binding IgG elevated moderately, we diagnosed him with AIHA. To our knowledge, there have been two reports that IVIG or corticosteroid were effective to HS [10,11]. Compared with those cases, our patient respond to IVIG and corticosteroid much better. These findings suggest that anemia in our patient is partially caused by immunological alteration as well as thrombocytopenia.

Since the complication of HS with AIHA or ITP has not been reported previously, it cannot be denied that these complications occurred coinsidentally. However, it is possible to supeculate some explanation for occurring AIHA or ITP with HS based on the some reason. First, a retrospective analysis of blood-bank records showed that out of 2618 patients who had a positive DAT or indirect anti-globulin test (IAT), 121 were identified with RBC autoantibodies; 41 of these patients had both allo- and autoantibodies to RBC antigens, whereas the remainder, 80, had only autoantibodies. At least 34 percent (12/41) of these patients developed their autoantibodies in temporal association with alloimmunization after recent blood transfusion [12]. Another report showed presence of both an anti-protein 4.2 antibody and other undefined autoantibodies against RBC associated with heavy transfusions in protein 4.2-negative HS patient [13]. Although it is unclear whether transfusion was related to production of autoreactive antibodies against RBC, it is suspected that activation of immune sysytem against both external and internal antigens was elicited by exposing alloprotein derived from transfused donor's RBC. Second, hypergammaglobulinemia may arise when specific helper T cells recognize B cells that have processed viral antigens irrespective of the B cell receptor specificity [14]. This deleterious role for nonspecific B cell activation by viral infection, arguing that it could potentially turn on anti-self-responses, may contribute to autoantibodies-associated hemolytic or thrombocytopenic manifestations. Moreover, Ward et al. has been reported that RBC-autoantigen-specific, interleukin-10-secreting regulatory T cell clones from a patient with autoimmune hemolytic anemia (AIHA), which had a functional phenotype [15]. Further careful observation is required for disclosing that this complication is not occurred incidentally.

## Competing interests

The authors declare that they have no competing interests.

## Authors' contributions

HY was responsible of the clinical management of the patient, acquisition of data, drafting the manuscript; HI was supervisor of clinical management of the patient and interpretation of data; TY, TI, AM were responsible of discussion and editing of the manuscript; TO was principal investigator of erythrocyte binding IgG quantitative analysis; MK was principal investigator of platelet specific autoantibodies. All authors read and approved the final manuscript.

## Consent

Written informed consent was obtained from the patient for publication of this case report and accompanying images. A copy of the written consent is available for review by the Editor-in Chief of this journal.
